# Association between vaginal microbiome alteration and povidone iodine use during delivery

**DOI:** 10.1186/s12866-023-03014-5

**Published:** 2023-11-17

**Authors:** Hongping Li, Hongqin Zhang, Linhua Geng, Hongli Huang, Chuan Nie, Yuanfang Zhu

**Affiliations:** 1 Shenzhen Children’s Hospital, Shenzhen, 518000 China; 2 Shenzhen Nanshan Maternity and Child Health Care Hospital, Shenzhen, 518000 China; 3 Baoan Maternal and Child Health Hospital, Jinan University, Shenzhen, 518000 China; 4 Shenzhen Luohu Maternity and Child Health Hospital, Shenzhen, 518000 China; 5 Guangdong Women and Children Hospital, Guangzhou, 510000 China

**Keywords:** Vaginal microbiota, Povidone iodine, Community state type, 16s rRNA sequencing

## Abstract

**Background:**

The vaginal microbiome is a dynamic community of microorganisms in the vagina. Its alteration may be influenced by multiple factors, including gestational status, menstrual cycle, sexual intercourse, hormone levels, hormonal contraceptives, and vaginal drug administration. Povidone iodine has been used before delivery to reduce infection that may be caused by the ascendance of pathogenic and opportunistic bacteria from the vagina to the uterus. This study aimed to elucidate the impact of povidone iodine use during delivery on the vaginal microbiome.

**Methods:**

This study enrolled a total of 67 women from maternity services in three hospitals. During the delivery process, we have applied povidone iodine in three doses such as low dose, medium dose, and high dose based on the amount of povidone iodine administered, thus, we studied the three groups of women based on the doses applied. Vaginal swab samples were collected both before and immediately after delivery, and the microbial communities were characterized using 16 S rRNA sequencing. The identification of differentially abundant microbial taxa was performed using ZicoSeq software.

**Results:**

Before delivery, the vaginal microbiome was dominated by the genus *Lactobacillus*, with different percentage observed (86.06%, 85.24%, and 73.42% for the low, medium, and high dose groups, respectively). After delivery, the vaginal microbial community was restructured, with a significant decrease in the relative abundance of *Lactobacillus* in all three groups (68.06%, 50.08%, and 25.89%), and a significant increase in alpha diversity across all 3 groups (*P* < 0.01). Furthermore, as the dose of povidone iodine used during delivery increased, there was a corresponding decrease in the relative abundance of *Lactobacillus* (*P* < 0.01). Contrary, there was an increase in microbial diversity and the relative abundances of *Pseudomonas* (0.13%, 0.26%, and 13.04%, *P* < 0.01) and *Ralstonia* (0.01%, 0.02%, and 16.07%, *P* < 0.01) across the groups. Notably, some functional metabolic pathways related to sugar degradation were observed to have significant change with increasing use of povidone iodine.

**Conclusion:**

Povidone iodine was associated with the vaginal microbiome alterations after parturition, and its significant change was associated to the dosage of povidone iodine administered. The escalation in iodine dosage was linked to a decrease in *Lactobacilli* abundance, and elevated prevalence of *Pseudomonas* and *Ralstonia*. There is a need for longitudinal studies to clearly understanding the effect of povidone iodine use on maternal and infant microbiome.

## Background

The microbial community inhabiting the vagina plays a fundamental role in women’s vaginal health, and it is influenced by various factors such as ethnicity or race, pregnancy, sexual intercourse, gestational status, menstrual cycle, menopause, contraceptive use, hormone levels, and more [1–5]. A healthy vaginal microbiome is typically dominated by genus *Lactobacillus*, and accumulating evidence indicates that the alterations of vaginal microbiome are associated with adverse health outcomes including vaginal infections, urinary tract infection, sexually transmitted diseases [6–9]. Gestational age is linked to the abundance of *Lactobacillus* species in the vaginal microbiome, accompanied with the significant reduction of microbial diversity [10, 11]. The derangement of the vaginal microbiome during pregnancy is reported as a contributor to adverse pregnancy and birth outcomes such as spontaneous abortion [12] and preterm birth [13].

The vaginal microbiome has been found to cluster into five community state types (CSTs) based on high-throughput sequencing in several cross-sectional and longitudinal cohorts [11, 14–17]. Four of these CSTs are dominated by different species of *Lactobacillus* (*L. crispatus*, CST I; *L. gasseri*, CST II; *L. iners*, CST III, and *L. jensenii*, CST V), whereas CST IV is heterogeneous and typified by a low abundance of *Lactobacillus* and a higher proportion of obligate anaerobic bacteria, including *Gardnerella*, *Prevotella*, *Atopobium, Megasphaera, Sneathia*, and *Streptococcus*. Vaginal microbial community has been showed as a dynamic microenvironment. Pregnancy leads to great stability of vaginal microbiome with the increase of relative abundance of *Lactobacillus* and decrease of the microbial alpha diversity, although, hormonal changes may influence the abundance of some pathogenic and opportunistic microbes and leads to adverse maternal health outcomes [10, 16]. At the time of labor onset, the vaginal microbiome is dominated by family *Lactobacillaceae* and *Bifidobacteriaceae* [18], and exposure to maternal vaginal fluids at birth with sterile gauz can partially restore the gut, oral, and skin microbiome of infants born via cesarean Sect. [19]. During the postpartum period (almost 6 weeks), the vaginal microbiome is characterized by poor *Lactobacillus* abundance and abundant vaginosis-associated bacteria [11, 17]. The relative abundance of *Lactobacillus* decreased and the microbial alpha diversity increased immediately after delivery was observed and linked to the povidone iodine administration for perineal disinfection during the delivery [20]. As a broad spectrum antiseptic for preventing skin infection, povidone iodine is used to clean and disinfect the vulva during delivery in maternity centers but its effect to the vaginal microbiome was not enough studied in China [21]. This study was carried out to investigate the association between povidone iodine use during delivery and vaginal microbial community alteration after delivery.

## Methods

### Study subjects

This study recruited a total of sixty-seven healthy women with gestational age (GA) greater than 37 weeks who delivered vaginally in maternity centers. The participants who did not accept to sign the consent for participation were excluded from the study. We have also excluded participants with antibiotic intake and amicrobial therapy during pregnancy, participants with probiotic intake within three months before delivery, participants with adverse pregnancy outcomes, participants with history of smoking and alcohol consumption during pregnancy, participants with complicated singleton pregnancy, and participants with newborn physical abnormalities. The participants were distributed as follows, twenty-seven women were recruited from Shenzhen Nanshan Maternity and Child Health Care Hospital (group LD, low dose) and received a personally administered 0.1% povidone iodine solution of 500 ml; thirteen women were recruited from Shenzhen Luohu Maternity and Child Health Hospital (group MD, medium dose) and received 250 ml of a 0.1% povidone iodine solution personally before entering the labor, delivery and recovery (LDR) room, and 250 ml of a 0.5% povidone iodine solution personally after entering the room; twenty-seven women were recruited from Shenzhen Baoan Maternal and Child Health Hospital (group HD, high dose) and received a personally administered 0.5% povidone iodine solution of 500 ml. Written informed consent was obtained from all study participants, and we have received ethics approval from the three hospitals separately.

### Sample collection

The vaginal swab samples were collected from all study participants in two different phases: the time of admission to the hospital for delivery (phase BD, before delivery), and immediately after delivery (phase AD, after delivery). The three sterile cotton swab sticks were used to collect vaginal swab samples by trained nurses to avoid insufficient DNA concentration. The sterile swab was placed carefully on the vaginal sidewall about halfway between the introitus and the cervix following previous reported method [20], rolled dorsally-ventrally back and forth four times to coat the swab, and all samples were stored at -80 °C storage after collection until DNA extraction.

### Microbiome profiling

Microbial DNA was extracted using QIAamp DNA Mini kit, and its concentration and purity were measured using the NanoDrop One (Thermo Fisher Scientific, MA, USA). V3-4 variable region of 16S rRNA gene was amplified using forward primer 338F (5’-ACTCCTACGGGAGGCAGCAG-3’), and reverse primer 806R (5’-GGACTACHVGGGTWTCTAAT-3’) [22]. PCR reactions, containing 25 µL 2× Premix Taq (Takara Biotechnology, Dalian Co. Ltd., China), 2 µL of each 10 mM primer and 3 µL DNA template in a volume of 50 µL, were amplified under the following thermal profile: 94 ℃ for 5 min, then 30 cycles of 94 ℃ for 30 s, 52 ℃ for 30 s, 72 ℃ for 30 s, followed by 72 ℃ for 10 min. PCR products were then pooled in equimolar and paired-end sequenced on an Illumina MiSeq platform with V3 chemistry.

The 16 S rRNA gene sequences were analyzed using the bioinformatics software package QIIME2 (version 2021.8) [23]. Paired-end reads were firstly denoised by QIIME2 with command “qiime dada2 denoise-paired”, aimed to merge paired-end reads, quality filtering, and to exclude chimeric and phiX sequences. Taxonomic assignment was performed against Greengenes (13_8 revision) database using command “qiime feature-classifier classify-sklearn”. Meanwhile, an array of alpha- and beta-diversity measures was generated using commands “qiime phylogeny align-to-tree-mafft-fasttree” and “qiime diversity core-metrics-phylogenetic”. In addition, functional metabolic pathways of microbiome were analyzed using software PICRUSt2.0 [24].

### Statistical analysis

Continuous variables were presented as the mean ± standard deviation (SD), and categorical characteristics were reported as numbers (percentages, %). All comparisons were performed in R software at a significance level of 0.05, using chi-square (Kruskal-Wallis test) and *t*-tests (ANOVA analysis) for categorical and continuous variables, respectively.

Principal coordinate ordination analysis (PCoA) was performed on weighted UniFrac distance, and accompanied with permutational multivariate analysis of variance (PERMANOVA, 999 permutations) with R package “vegan”. To analyze the significant differences in microbes between two groups with adjusted *P* < 0.05, ZicoSeq software was used [25].

## Results

### Demographic and clinical data

Demographic and clinical characteristics of the study subjects and their newborns were provided in Table 1. A total of 67 healthy and asymptomatic women were recruited, including 27 from the low dose (LD) group, 13 from the medium dose (MD) group and 27 from the high dose (HD) group. All participants were of Han ethnicity and had a mean age of 30.0 years, with an average pre-pregnancy BMI of 20.0 kg/m^2^. All women gave birth within the gestational range of 37th and 42nd week, with an average gestational weight gain of 14.28 kg. The neonatal birth weight was also recorded, with an average of 3282.4 g and a gender distribution of 36 boys and 31 girls. There was no significant difference observed in these characteristics between the three groups.

**Table 1:** Demographic and clinical data for study participants

Characteristics	Low dose (LD, n = 27)	Medium dose (MD, n = 13)	High dose (HD, n = 27)	P value
Mother’s age, yrs mean (SD)	30.5 ± 3.1	30.2 ± 4.1	28.6 ± 2.7	0.082
Mother’s pre-pregnancy BMI, kg/m^2^ mean (SD)	20.2 ± 2.1	20.6 ± 2.2	19.5 ± 2.3	0.274
Gestational weight gain, kg mean (SD)	14.9 ± 4.5	13.2 ± 3.8	14.26 ± 0.37	0.465
Gestational week, wks mean (SD)	39.9 ± 0.9	39.6 ± 0.8	39.5 ± 1.0	0.287
Neonatal’s birth weight, g mean (SD)	3299 ± 306	3406 ± 243	3206 ± 289	0.12
Neonatal’s gender (male/female)	12/15	9/4	15/12	0.509

### Comparisons of microbial community between the 3 groups

**Fig. 1:**
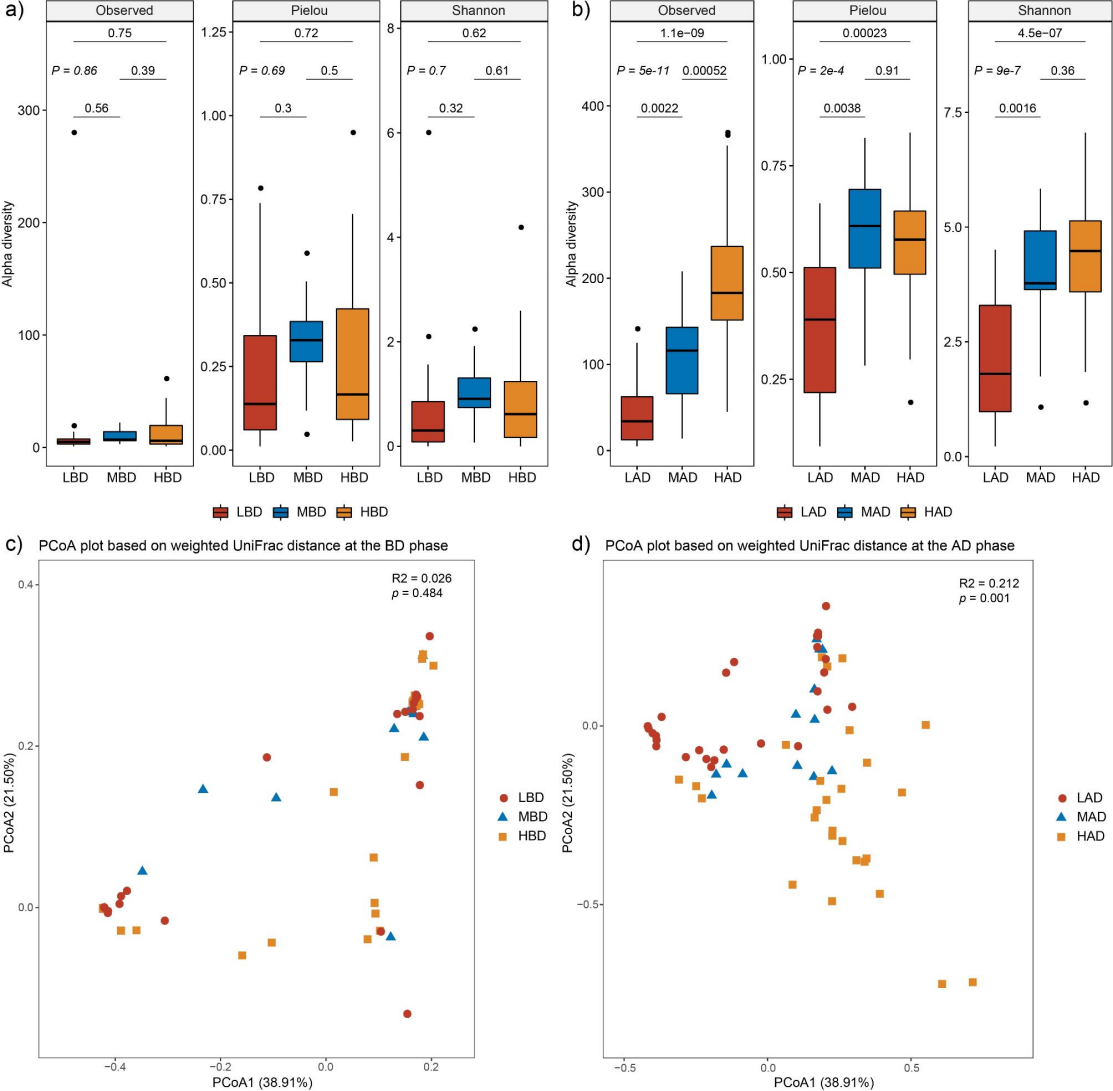
The comparisons of the alpha and beta diversity. **(a)** Alpha diversity plots in microbial community and the comparisons between the 3 groups at the BD phase. **(b)** Alpha diversity plots in microbial community and the comparisons between the 3 groups at the AD phase. **(c)** PCoA analysis of 3 groups based on weighted UniFrac distance at the BD phase. **(d)** PCoA analysis of 3 groups based on weighted UniFrac distance at the AD phase

In this study, alpha diversity was represented using observed feature number, Pielou index, and Shannon index. Prior to delivery, the average values of Shannon index (0.74 for the LBD group, 1.03 for the MBD group, and 0.90 for the HBD group, *P* = 0.7), Pielou index (0.26 for the LBD group, 0.33 for the MBD group, and 0.28 for the HBD group, *P* = 0.69), and observed feature number (15.74 for the LBD group, 9.62 for the MBD group, and 12.41 for the HBD group, *P* = 0.86), there was no significant difference between the three groups, respectively (Fig. 1a). After delivery, the mean difference was statistically significant in Shannon index (2.03 for the LAD group, 3.08 for the MAD group, and 4.27 for the HAD group, *P* < 0.01), Pielou index (0.38 for the LAD group, 0.57 for the MAD group, and 0.56 for the HAD group, *P* < 0.01), and observed feature number (45.22 for the LAD group, 106.31 for the MAD group, and 192.63 for the HAD group, *P* < 0.01) between the three groups, respectively (Fig. 1b).

During the AD phase, there was a significant increase in Shannon index (LD, *P* < 0.01; MD, *P* < 0.01; HD, *P* < 0.01), Pielou index (LD, *P* = 0.057; MD, *P* < 0.01; HD, *P* < 0.01), and observed feature number (LD, *P* = 0.025; MD, *P* < 0.01; HD, *P* < 0.01) across all three groups, as compared to the BD phase.

To compare the overall vaginal microbial community between the three groups, principal coordinate analysis was implemented on the weighted UniFrac distance. The results showed that vaginal samples before delivery from the three groups clustered together according to PERMANOVA analysis (R^2^ = 0.026, *P* = 0.484, Fig. 1c). After delivery, the samples were significantly different and separated (R^2^ = 0.212, *P* = 0.001, Fig. 1d). The vaginal microbial community showed the significant differences between the BD and AD phase in all three groups separately (LD, R^2^ = 0.175, *P* = 0.001; MD, R^2^ = 0.323, *P* = 0.001; HD, R^2^ = 0.279, *P* = 0.001; data not shown).

### Taxonomic compositional differences between the 3 groups

**Fig. 2:**
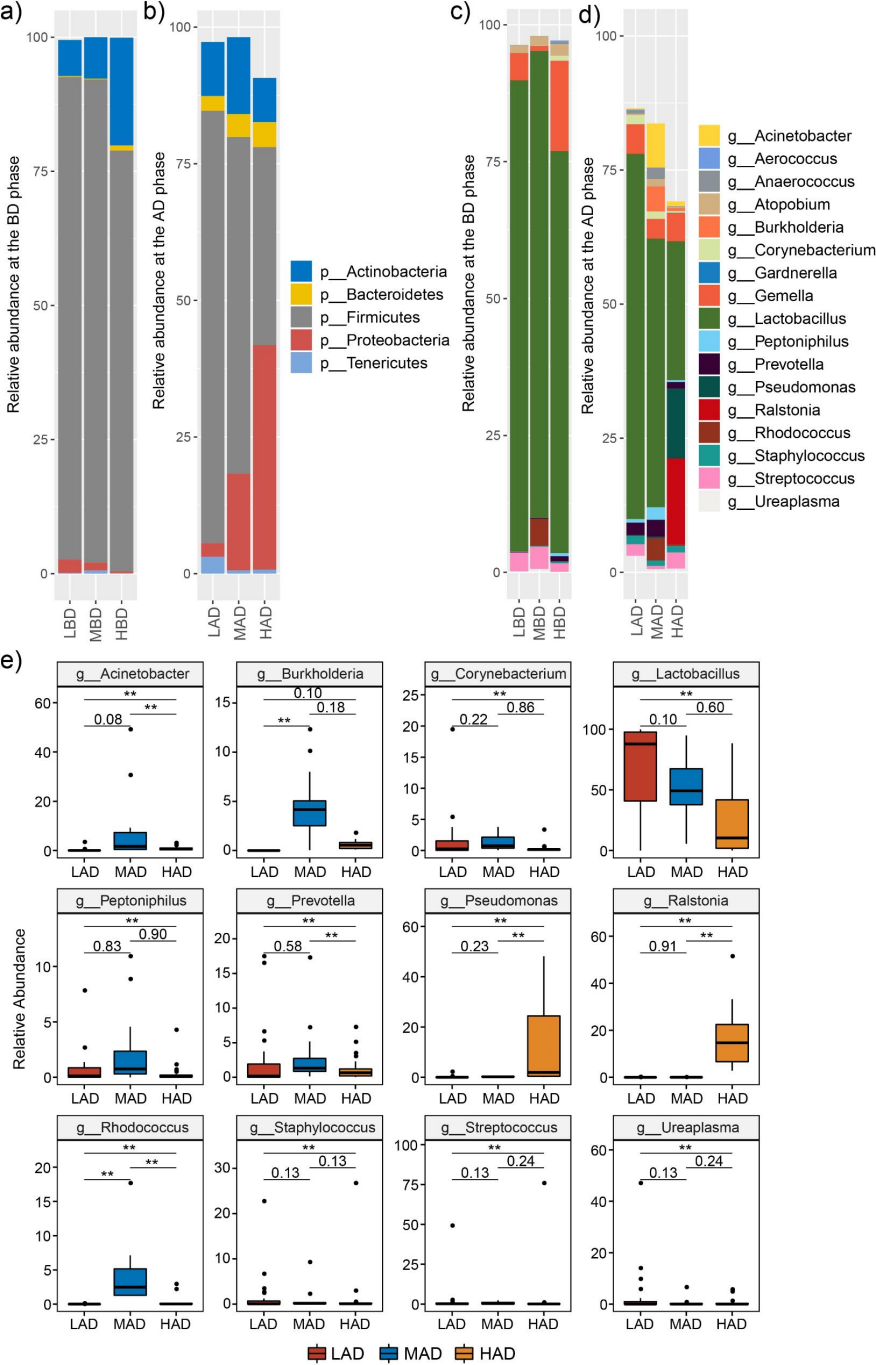
Relative abundances and differences of abundant microbes between the three groups. **(a)** Relative abundances of the abundant phyla at the BD phase. **(b)** Relative abundances of the abundant phyla at the AD phase. **(c)** Relative abundances of the abundant genera at the BD phase. **(d)** Relative abundances of the abundant genera at the AD phase. (e) The comparisons of the genus at the AD phase

The composition of the vaginal microbial community was characterized by high-throughput sequencing of the 16 S rRNA V3-V4 high-variable region. A total of 6,545,489 sequence reads from 134 vaginal samples were included in the analysis. The average sequence read count was 48,847 per sample, with a median of 48,207, and the mean and median read lengths were 420 and 423 bp, respectively. The total ASVs (amplicon sequence variants) was 13,643, it increased from LD (2,168) to MD (2,373) and HD (10,250) groups. Rarefaction curves suggested that the sequencing depth was sufficient (data not shown). The average relative abundances of the five most abundant phyla (*Actinobacteria*, *Bacteroidetes*, *Firmicutes*, *Proteobacteria*, and *Tenericutes*) were shown in Fig. 2a and b. The results demonstrated that during pregnancy, the vaginal microbial community was largely dominated by phyla *Firmicutes* (average, 89.89% of the LBD group, 90.0% of the MBD group, and 78.43% of the HBD group) and *Actinobacteria* (average, 6.69% of the LBD group, 7.70% of the MBD group, and 20.06% of the HBD group), accounting for more than 96% of the microbial population across all three groups. The substantial shifts in bacterial phylum structure were observed after delivery, with dramatically decreased proportions of *Firmicutes* (79.08% of the LAD group, 61.57% of the MAD group, and 36.27% of the HAD group) and increased abundances of *Proteobacteria* (2.51% of the LAD group, 17.70% of the MAD group, and 41.04% of the HAD group). The low decrease in the relative proportion of *Firmicutes* was observed in the LD group, while the high decrease was observed in the HD group.

The abundant genera were *Acinetobacter*, *Aerococcus*, *Anaerococcus*, *Atopobium*, *Burkholderia*, *Corynebacterium*, *Delftia*, *Gardnerella*, *Lactobacillus*, *Peptoniphilus*, *Prevotella*, *Pseudomonas, Ralstonia*, *Rhodococcus*, *Staphylococcus*, *Streptococcus*, and *Ureaplasma* (Fig. 2c and d). The genus *Lactobacillus*, an important genus in the *Firmicutes* phylum, was found in 128 out of 134 vaginal samples, with a mean proportion that decreased from 86.06% before delivery to 68.06% after delivery in the LD group, from 85.24% decreased to 50.08% in the MD group, and from 73.42% decreased to 25.89% in the HD group. Among the 3 groups, the relative proportion of *Lactobacillus* decreased with the increasing usage of povidone iodine. The significant decrease was observed from the LD group to the HD group.

To test for the significant differences in the relative abundances of genera, ZicoSeq software with multiple corrections was used [25], accounting for over 1% in at least one group. There was no significant difference among the three groups before delivery. The significant difference was observed among the three groups after delivery, as shown in Fig. 2e. Compared to the LAD group, genera *Burkholderia*, and *Rhodococcus* increased significantly in the MAD group, and genera *Acinetobacter*, *Pseudomonas*, *Ralstonia*, *Rhodococcus*, and *Streptococcus* increased significantly in the HAD group. Genera *Corynebacterium*, *Lactobacillus*, *Peptoniphilus*, *Prevotella*, *Staphylococcus*, and *Ureaplasma* decreased significantly in the HAD group. Compared to the MAD group, the average abundance values of genera *Acinetobacter*, and *Prevotella* were significantly lower, whereas *Pseudomonas* and *Ralstonia* were significantly higher in the HAD group. The increased usage of povidone iodine was associated with a decrease in the average relative abundance of *Lactobacillus*, from 68.06% in the LAD group to 50.08% in the MAD, and further to 25.89% in the HAD group. The average relative abundances of *Pseudomonas* and *Ralstonia* increased from the LAD group (0.13% and 0.01%), to the MAD (0.26% and 0.02%) and HAD groups (13.04% and 16.07%).

### Different functional metabolic pathways between the 3 groups

**Fig. 3:**
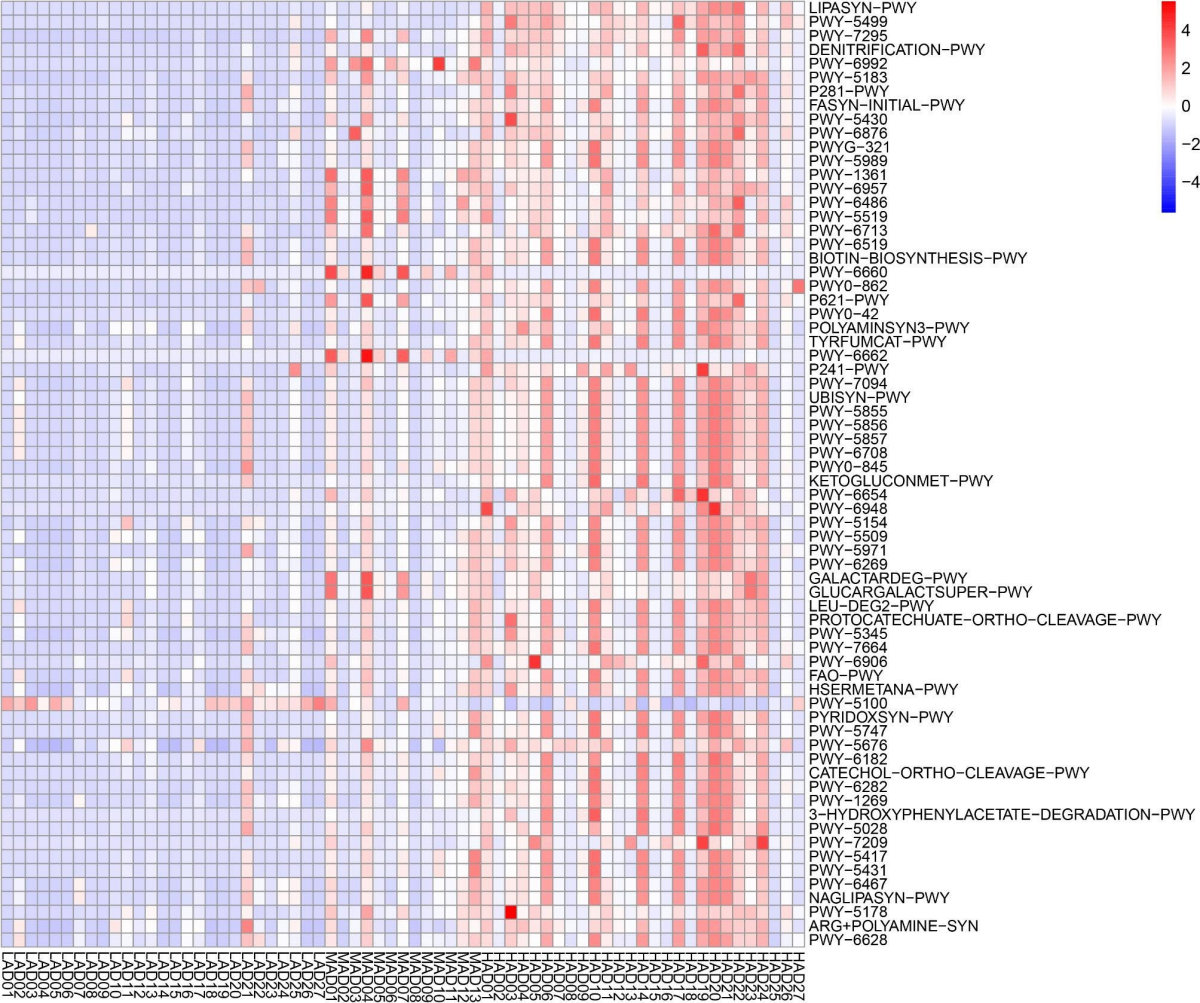
Different functional metabolic pathways between the 3 groups at the AD phase

The functional metabolic pathways of vaginal microbial community were analyzed against the MetaCyc database using the software PICRUSt2.0 [24]. ANOVA with Bonferroni correction at the 0.01 level was used to test for the significant difference in metabolic pathways. The results revealed that there was no significant difference observed between metabolic pathways at the phase of before delivery. After delivery, 68 metabolic pathways were found to differ significantly between the 3 groups (Fig. 3). Of these, 27 pathways were related to the sugar degradation (arabinose, anhydrofructose, and rhamnose), amino acid (L-tyrosine, L-leucine, and L-histidine), vitamin B6, hydroxyphenylacetate, and benzoyl-Co et al.; 30 were involved in the biosynthesis of fatty acid, amino acid (L-phenylalanine, arginine, and L-methionine), ubiquinol, pyridoxal, and biotin et al. Almost all these pathways significantly increased in the HAD group when compared to the LAD group. These findings suggested that the metabolic pathways of the vaginal microbial community after delivery were significantly influenced by the dose of povidone iodine used.

## Discussion

This study aimed to investigate the association between the povidone iodine use during the process of delivery and vaginal microbiome. The vaginal microbial alteration was observed immediately after delivery in low, medium, and high dose groups separately, and was characterized by a significant decrease in *Lactobacillus* and an increase in alpha diversity. A dose-dependent relationship was established between povidone iodine administration and the relative abundance of *Lactobacillus*, with higher doses associated with lower levels of *Lactobacillus*. An increased microbial diversity and elevated relative abundances of *Pseudomonas* and *Ralstonia* were observed with escalating doses of povidone iodine. Consistent with the previous study that reported a significant increase in microbial diversity and an altered community structure characterized by reduced *Lactobacillus* abundance in vaginal samples immediately after delivery [20]. These findings were also consistent with previous investigations of the vaginal microbial community at postpartum 6 weeks, which reported higher alpha-diversity, increased relative abundance of *Pseudomonas*, and a pronounced decrease in the abundance of genus *Lactobacillus* compared to the pregnancy period [11, 17, 26]. Before delivery, there were no differences in the microbial diversity (represented by observed feature number, Pielou index, and Shannon index) and community (measured by weighted UniFrace distance) between the three groups. However, significantly increased microbial diversity and changed community were observed between the three groups immediately after delivery. Moreover, the observed feature number and Shannon index increased with the increasing use of povidone iodine, indicating an increase in the variety of bacteria.

The vaginal microbiome forms the first barrier against the external environment, and it is well known that a vaginal microbiota is dominated by genus *Lactobacillus*, which plays an important role in protecting the host from various fungal, bacterial, and viral pathogens [27]. During pregnancy, normal vaginal microbiome is often dominated by *Lactobacillus* [3] due to the changes of hormone levels, especially estrogen [28]. *Lactobacillus spp.* in the vagina can produce lactic acid and other organic acids to reduce the vaginal pH value by metabolizing glycogen [29]. A low pH value protects against viral, bacterial, and parasitic agents [2]. Furthermore, *Lactobacillus spp.* can produce hydrogen peroxide, bacteriocin and biosurfactants, which appear to ensure normal vaginal microbiota and effectively inhibit the colonization of pathogens [1]. However, in this study, the relative abundance of genus *Lactobacillus* decreased with increasing use of povidone iodine during delivery, while *Ralstonia* and *Pseudomonas* increased. This change might disrupt the natural protective role of *Lactobacillus* and lead to an increase in the pH value in the vagina, thereby increasing the risk of vaginal infections. A low abundance of *Lactobacillus* species and an increase in facultative and anaerobic organisms were also associated with a high risk of sexually transmitted diseases [27], and bacterial vaginosis [7]. Moreover, a low *Lactobacillus* species abundance in the vaginal microbiome is associated with a higher risk for preterm birth [30]. *Pseudomonas*, in particular, has been observed to be significantly enriched in the vaginal microbiome of women with recurrent spontaneous abortion [12, 31], and positively associate with high-risk human papilloma virus (hrHPV) infection [32]. Jeff et al. conducted a study investigating the influence of infectious factors on cervical cancer, utilizing *Pseudomonas aeruginosa* as a bacterial agent and *Lactobacillus* as a control [33]. Their investigation revealed an up-regulation of integrins expression in cervical cancer tissues, and notably, *Pseudomonas aeruginosa* was found to enhance integrin expression in cervical cancer cell lines, while the *Lactobacillus* control group showed no change. These findings suggest a potential role of *Pseudomonas* in promoting the development of cervical lesions. Opportunistic pathogen *Ralstonia spp.* is also becoming more and more prevalent in cases of infection [34]. *R. pickettii* exhibits a high frequency of occurrence within the HPV-positive group [35]. Zhang et al. further found a significant abundance of *R. pickettii* in the vaginal microbiome women infected with HPV, while the relative abundance of *Ralstonia* was comparatively less prevalent in HPV-infected and cervical intraepithelial neoplasia diagnosed women [36]. These changes in vaginal microbiome with reduced *Lactobacillus* abundance and increased opportunistic organisms might predispose the host to increased risk of bacterial infection. Further comprehensive investigations are imperative to elucidate the underlying mechanisms.

Furthermore, the deposition of glycogen is known to support the proliferation of *Lactobacillus*, which in turn metabolizes glycogen to lactic acid, thereby leading to a low pH environment [29]. However, our findings showed that several metabolic pathways associated with sugar degradation (such as PWY-7295: L-arabinose degradation IV; PWY-6992: 1,5-anhydrofructose degradation; PWY-6486: D-galacturonate degradation I; PWY-5519: D-arabinose degradation III; PWY-6713: L-rhamnose degradation II, et al.) significantly increased in the HAD group. These increases might accelerate the consumption of glycogen, leading to a failure in maintaining the growth of *Lactobacillus* and acid production. In addition, PWY-7295 and PWY-6992 were also more enriched in women with preterm premature rupture of membranes [37], and they both significantly increased in the HAD group. The results of functional metabolic pathway also showed dramatic changes in the vaginal microbiome with increasing use of povidone iodine. As the functionality of a microbial community is typically studied through metabolic profiling, which involves the measurement of small molecules such as carbohydrates and amino acids, further research with metabolomics profile is required to confirm the observed changes in metabolic function.

Although the present study provided important insights into the effect of povidone iodine use during the delivery process on the vaginal microbiome, there were several limitations that should be acknowledged. Firstly, the cross-sectional design of this study is insufficient to establish causality or confirm the generalizability of the findings to different populations. Therefore, further research employing larger and more diverse cohorts from different geographic regions is necessary to validate the current observations and draw more robust conclusions. Secondly, the use of the V3-V4 region of 16S rRNA to profile the vaginal microbiome community in this study differs from previous investigations that employed the *cpn60* gene. This discrepancy in the gene target selection may introduce variations in the microbial composition analysis. Future investigations should consider exploring the lower taxonomic levels using whole genome sequencing, which would minimize the potential biases associated with the amplified method and provide a more comprehensive understanding of the vaginal microbial community. Lastly, assessing the functionality of the microbial community often involves analyzing the metabolic profiles of small molecules, such as carbohydrates and aminio acids. Incorporating metabolomics approaches in future research is warranted to gain a deeper understanding of the funcional changes occurring within the vaginal microbial community. This would provide valuable insights into the potential metabolic interactions and their implications in the context of povidone iodine use during the delivery process.

In conclusion, this study demonstrated that the use of povidone iodine during delivery is associated with significant changes in the vaginal microbiome, as evidenced by reduced relative abundance of *Lactobacillus* and increased presence of some pathogenic bacteria. The current findings provide valuable insights into the community profile of the vaginal microbiome immediately after delivery and its relationship with povidone iodine use.

## Data Availability

Sequence data and metadata for each sample have been deposited in the NCBI Sequence Read Archive database (BioProject ID: PRJNA1014502).
